# Thermal and Structural Behavior of Investment Casting Molds Modified with Biodegradable Walnut Shell Chips

**DOI:** 10.3390/ma18184289

**Published:** 2025-09-12

**Authors:** Marcin Małek, Janusz Kluczyński, Rafał Grzejda, Paweł Wiśniewski, Agnieszka Jenerowicz, Ireneusz Ewiak

**Affiliations:** 1 Institute of Civil Engineering, Faculty of Civil Engineering and Geodesy, Military University of Technology, 00-908 Warsaw, Poland; 2 Institute of Robots & Machine Design, Faculty of Mechanical Engineering, Military University of Technology, 00-908 Warsaw, Poland; janusz.kluczynski@wat.edu.pl; 3 Faculty of Mechanical Engineering and Mechatronics, West Pomeranian University of Technology in Szczecin, 70-310 Szczecin, Poland; rafal.grzejda@zut.edu.pl; 4 Faculty of Materials Science and Engineering, Warsaw University of Technology, 02-507 Warsaw, Poland; pawel.wisniewski@pw.edu.pl; 5 Department of Imagery Intelligence, Faculty of Civil Engineering and Geodesy, Military University of Technology, 00-908 Warsaw, Polandireneusz.ewiak@wat.edu.pl (I.E.)

**Keywords:** casting molds, precision casting, gas permeability, corundum, binders, nano-SiO_2_, walnut shell chips

## Abstract

Two types of spherical mold samples—designated PW1 (reference) and PW2 (modified) were prepared using the dip-and-sprinkle method. Both samples consisted of seven layers, but PW2 was differentiated by the incorporation of 5 wt.% ground walnut shell chips into the fifth layer of its structure. The aim of this modification was to assess the feasibility of employing biodegradable organic additives to generate controlled porosity after thermal decomposition, thereby enhancing gas transport through the mold structure. The gas permeability of the samples was determined across a broad temperature range from 25 to 950 °C using a dedicated, custom-built test rig developed for elevated-temperature permeability assessments. The results revealed that the inclusion of walnut shell chips significantly increased the gas permeability of the molds by approximately 42% at ambient temperature and 36% at 950 °C, attributable to the formation of stochastically distributed macro-voids upon burnout of the organic additive. The study demonstrates that selective layer modification using natural waste materials can be a viable method for tailoring functional properties of ceramic molds, offering a cost-effective, sustainable, and easily scalable alternative to conventional pore-forming strategies.

## 1. Introduction

The lost-wax investment casting process has been practiced for millennia and remains a cornerstone of manufacturing complex, high-performance metal components [1,2,3,4]. It saw significant advancement around the mid-20th century—notably during and after World War II—when the aerospace and power generation industries drove rapid developments in precision casting technology [1,2,3,4,5,6]. Today, this process is indispensable for producing critical turbine engine parts that demand exceptional dimensional accuracy [7,8,9] and material integrity [10,11]. Such cast components, used in both aircraft engines and energy turbines, must endure extreme operating conditions. Therefore, investment casting is engineered to yield near-net-shape parts with excellent surface finishes (as fine as ~5 µm Ra) and complex geometries [12,13]. Moreover, advanced casting techniques are employed to tailor the alloy microstructure for maximal high-temperature strength and creep resistance [7,8,9,10,11,14,15]. These achievements are made possible by multi-layer ceramic shell molds with carefully designed structures and properties [7,8,9,10,11,14,15]. The mold not only forms the final shape with high fidelity, but also controls the solidification rate by extracting heat from the molten alloy in a prescribed manner, thereby influencing grain structure [7,8,9,10,11,14,15]. Over the decades, numerous refinements to shell mold materials and construction have been introduced to meet the increasing quality requirements of aerospace and energy castings [2,10,11,15]. Despite these advances, ceramic shells still face challenges (e.g., maintaining strength, thermal stability, and permeability) that require ongoing research and innovation [10,11].

Modern ceramic shell molds are typically made of high-purity refractory materials—often alumina (corundum) as the primary flour [16]—combined with a colloidal silica [17] binder and various additives [18]. Corundum (α-Al_2_O_3_) is favored for face coats and backup layers due to its high melting point [19], chemical inertness, and stability [20], which help prevent metal–mold reactions [21] and erosion by the molten alloy [22,23,24,25]. Other refractories, such as aluminosilicates, zircon, fused silica [26,27], or SiC [28], may be used in specific layers [29,30,31] to achieve the desired thermal or chemical characteristics [32,33]. The binder is usually a water-based silica sol (e.g., LUDOX AM30 (Grace, Columbia, MD, USA) containing ~30 wt.% nano-SiO_2_), which yields a slurry that coats the wax pattern uniformly and later hardens into a ceramic matrix upon drying and firing [16,17,18,25]. Compared to older ethyl silicate or organic binder systems, colloidal silica is relatively eco-friendly and does not produce toxic fumes during shell preparation or firing [5,6,26]. The slurry formulation often includes rheology modifiers, anti-foaming agents, and wetting agents to ensure proper flow and coating coverage [16,17,18,30]. The particle size distribution of the ceramic flour is carefully chosen—fine powders for face coats to capture detail, and coarser grains for intermediate and backing layers—to optimize packing density and minimize defects [16,17,18,29]. A broad particle size range can improve packing and reduce large voids, but excessive fines can increase slurry viscosity and reduce permeability [29,30]. Therefore, achieving a balance in slurry rheology and stability is crucial: it must allow easy dipping and draining while preventing sedimentation or agglomeration [16,17,18,30]. Indeed, the flow behavior of such ceramic slurries has been the subject of extensive study, as it directly affects the uniformity and strength of each shell layer [30]. In some cases, additives are introduced to enhance slurry performance—for example, small amounts of polymer binders—like poly (vinyl alcohol)—can improve green strength and coat adherence [23], and short fibrous reinforcements (ceramic or polymer fibers) have been explored to increase crack resistance or handle thermal stress [18,24]. For highly reactive alloys (such as titanium aluminides), specialized face coat systems have been developed (e.g., TiAlO-based or zirconia-based slurries with tailored binders) to prevent any reaction between the metal and mold [32,33]. These material considerations ensure that each layer of the shell mold fulfills its role: the primary (face coat) layers capture fine details and resist molten metal attack, while the subsequent backup layers build thickness for strength, and the outermost layers provide structural rigidity. All layers together form a robust ceramic shell capable of containing the metal during casting and influencing its solidification behavior.

Ceramic shells are built in stages on expendable wax or polymer patterns using the conventional dip-and-sprinkle method [16,17,18,19,20,21]. First, the cleaned wax pattern is immersed in the fine-grained primary slurry (the investing coat), then withdrawn and allowed to drain off the excess. While the coating is still wet, a rain of refractory stucco particles (e.g., fine corundum sand) is sprinkled or fluidized onto it, which adheres and builds up the layer thickness [16,17,18,22]. This creates a face layer with a coarse outer texture that promotes good adhesion with the next layer. After air drying under controlled conditions, the process is repeated with subsequent layers using coarser slurries and larger stucco grains for the intermediate and structural coats [16,17,18,22]. Typically, an investment shell for high-performance castings consists of a face coat and one or two fine sealing layers, followed by several strengthening layers, yielding a total of 5–10 layers depending on part size and requirements [16,17,18,19,20,21]. The final layer is often a seal coat without stucco, intended to cover any exposed stucco and give a smooth outer surface. Each layer must be thoroughly dried before the next is applied; this drying is carried out in a controlled environment (temperature and humidity) to prevent rapid evaporation that could cause cracking or delamination [19,20,21]. It is common practice to dry shells in a conditioned room or chamber, ensuring gentle solvent evaporation (for water-based binders) and minimizing thermal gradients [19,20,21]. Once the multilayer shell is completed and dried (often after a curing period of several days), the wax pattern is removed. Wax elimination is typically achieved through autoclave steam dewaxing, where high-pressure steam melts and evacuates the bulk of the wax, leaving a hollow ceramic shell [22,23,24,25]. Any residual pattern material is burned out during the subsequent firing step. The ceramic shell is then fired at high temperature (typically 800–1100 °C, depending on the materials) to sinter the binder and strengthen the shell to casting [22,23,24,25]. A slow heating schedule is used to avoid thermal shock; this fire both solidifies the shell’s structure and removes any remaining organic matter or moisture. An optional additional heat treatment (annealing) may be applied to tune the shell’s mechanical properties or relieve stresses. The result is a rigid, porous ceramic shell mold ready to receive molten metal. Pouring of the superalloy is often performed under vacuum or inert atmosphere (especially for aerospace castings) to prevent oxidation. The ceramic shell must withstand the metallostatic pressure and thermal load of the liquid alloy without cracking, all while allowing the alloy to solidify in the desired manner. After the metal cools and solidifies, the shell is broken away (knocked out), usually by mechanical vibration or water-blasting, to reveal the cast part. This entire fabrication sequence is labor- and time-intensive, and researchers continually seek improvements to reduce cycle time and cost (for example, spraying the slurry instead of dipping can speed up shell building in some cases [22], and novel binders have been introduced to shorten drying times [27]). Nonetheless, the traditional dip-and-stucco method remains predominant due to its reliability and the high quality of shells it produces.

The performance of a ceramic shell mold during casting depends on a careful balance of its physical and mechanical properties. The shell must have high hot strength and creep resistance to support the liquid metal and maintain dimensional accuracy at casting temperatures [22,23,24,25]. It must also resist thermal shock when the molten alloy (often over 1500 °C for Ni-based superalloys) is poured in, and survive until the metal solidifies. Equally important, the shell should be gas-permeable—that is, it should allow air and process gases to escape freely through its walls during mold filling and solidification [22,23,24,25]. If a shell is too impermeable, trapped air and vaporizing residues can cause internal pressure, leading to defects such as blowholes, gas porosity, or misruns in the casting [27,34]. On the other hand, excessive porosity or large pores in the shell can undermine its mechanical strength and allow metal penetration into the mold wall, which is undesirable. Thus, an optimal porosity is needed: fine interconnected pores that permit venting of gases but do not significantly weaken the structure [25,26,27,28,29]. The pore structure of the shell (pore size, distribution, and volume fraction) is primarily determined by the particle sizes of the refractory, the binder content, and any volatile additives that burn out during firing [26,27,28,29]. For example, using a broad, bimodal particle distribution can maximize packing density (reducing large voids) but may yield fewer, smaller pores [29]; conversely, introducing deliberately sacrificial fillers (like organic particles or fibers) creates additional voids after burnout, raising permeability [27,34,35]. The binder itself plays a crucial role, as demonstrated by Wiśniewski et al. [25], which shows that different binder formulations can result in significant differences in the porosity and gas permeability of the final shell. A silica sol binder that gels densely may produce a more tightly sealed ceramic matrix, whereas incorporating a polymeric or foam-forming binder could leave a more open porous network after firing [23,25]. Furthermore, chemical interactions at high temperatures can modify the pore structure—for instance, if silicon carbide fillers are used, they may react with an oxide binder or the furnace atmosphere to form gaseous species or secondary phases [28], potentially altering both the strength and permeability of the shell. The shell’s thermal conductivity is also influenced by its porosity: pores are insulative, so a higher porosity shell tends to slow down heat extraction from the solidifying metal [26,27,28,29]. In directional solidification casting, a certain level of insulation is beneficial for controlling cooling rates. However, in conventional casting, excessive insulation (or too thick shell) may prolong solidification and promote coarse grains or shrinkage defects [9,15,20]. Therefore, engineers must optimize shell thickness and material to achieve the desired thermal profile in the casting. All these factors underscore that controlling the microstructure of the ceramic shell (through material selection and processing) is just as crucial as controlling and maintaining the metal alloy’s solidification. Indeed, recent overviews highlight that modern ceramic shells must simultaneously satisfy often competing criteria—strength, permeability, thermal stability, dimensional precision, and chemical inertness—and this remains an active area of materials engineering research [10,11].

One persistent problem in investment casting is the evolution of gases when the hot metal comes into contact with the mold. Air within the mold cavity, vaporizing binders or pattern materials, and gases from any reactions must all escape through the ceramic shell’s porous network. Insufficient gas permeability can lead to pressure buildup and gas entrapment, causing defects in the casting [27,34]. Traditional shells made purely of dense ceramic (especially with fine-grained slurries) can be relatively impervious [27]. To address this, foundry engineers have developed strategies to tailor the permeability of shells without sacrificing their strength. One approach is to adjust the as-built porosity by modifying the shell recipe: for example, adding fugitive additives to the slurry that create pores upon burnout. Synthetic polymer additives have been explored—Sanjay and Karunakar demonstrated that incorporating a small amount of acrylonitrile–butadiene–styrene (ABS) plastic powder and needle coke (a carbonaceous filler) into the shell layers significantly increases the fired porosity and permeability, resulting in fewer gas-related casting defects [27]. However, purely synthetic additives can introduce residues or may not decompose cleanly. Recent research has therefore turned to naturally derived, biodegradable pore-formers that are both effective and environmentally benign [34,35]. Pattnaik and Sutar have investigated organic fillers, such as sawdust (wood flour), in the secondary layers of the shell, demonstrating that incorporating approximately 5 wt.% sawdust into the slurry can increase the shell’s porosity and double its gas permeability, while still retaining acceptable mechanical strength after firing [36]. The porous structure left by the burnt-out sawdust helped vent gases and even improved the feeding of castings, reducing internal defects [36]. In a similar vein, agricultural waste products such as walnut shell flour offer a promising option as pore-forming additives. Walnut shell is a lignocellulosic material that burns out almost completely during firing, leaving behind a network of micro-porosity. It is inexpensive and widely available as a by-product of the food industry, making it an attractive green additive. In the present work, this concept is explored by incorporating fine walnut shell particles into one of the intermediate mold layers. The goal is to increase gas permeability in that layer—effectively creating a venting network within the shell—to facilitate the escape of gases during pouring and solidification. Using a waste-derived additive, such as walnut shell, aligns with both environmental and economic objectives by reducing reliance on virgin materials and improving casting yield. An added benefit is that such bio-additives decompose into gaseous products (CO_2_, H_2_O, etc.) rather than harmful solid residues, and they contribute significantly less to pollution compared to synthetic substances [3,26]. Beyond additives in the slurry, other innovative techniques have emerged to enhance shell permeability. Another approach is the use of mechanical drilling or patterning to introduce vent holes in non-critical areas of the shell after it is built—for instance, ultrasonic micro-drilling has been used to perforate shells, yielding a significant permeability boost and reduced casting porosity [11]. However, such post-processing methods carry risks of shell damage and are less practical for production. A more practical strategy is to engineer the slurry composition itself for enhanced permeability: researchers have incorporated combustible wax-based powders into shells (similar to pattern wax), which melt or vaporize during firing, leaving behind additional porosity [35]. Pattnaik and Sutar demonstrated that adding 4 wt.% polyethylene wax powder to the secondary (back-up) coats resulted in a shell with higher green strength and approximately 20% higher fired porosity and permeability, which translated to fewer porosity defects in the cast product [37]. Notably, the minor decrease in fired flexural strength observed was acceptable, as the shell’s role is temporary and it is broken off after solidification [35]. Fiber additions can also influence permeability: ceramic fibers (which remain in the shell) primarily boost strength, whereas sacrificial polymer fibers (like nylon) burn away and leave elongated pores that may slightly increase permeability while also affecting mechanical properties [18,24]. For example, Huang et al. found that introducing a small fraction of nylon fibers alongside ceramic fibers into a silica sol shell improved the shell’s fracture toughness and did not excessively compromise permeability. However, an optimal fiber content had to be found [24]. Overall, these advanced techniques aim to create a shell that is just permeable enough to vent gases and prevent defects, yet is still strong and stable throughout the casting process.

Modern materials engineering of ceramic shells increasingly emphasizes sustainability, cost-effectiveness, and technical performance. The use of water-based binders (colloidal silica) instead of alcohol-based or chemical-setting binders has virtually eliminated the emission of noxious fumes and vastly improved working conditions in shell fabrication [3,26]. Likewise, incorporating recycled or renewable materials into shell construction can reduce waste and lower material costs. The idea of using walnut shell flour exemplifies this trend—it repurposes agricultural waste into a value-added component of the mold, enhancing permeability and then completely combusting away. Such bio-additive approaches not only improve casting quality but also reduce the environmental footprint of the process [3,34,35]. In parallel, researchers are looking at the end-of-life of ceramic shells themselves. Spent shells (after knockout) have historically ended up as landfill, but recent studies have explored the valorization of ceramic shell waste—for instance, crushing used shells to reuse them as refractory aggregate or as feedstock for other ceramic products [38]. Koralnik demonstrated a recycling route for alumina-based shell waste, showing it can be classified, ground, and reused in new molds or other ceramics without loss of performance [38]. This circular approach enhances the sustainability of precision casting by reducing raw material consumption and waste generation. Another avenue for improvement is to expedite the shell production cycle, thereby saving energy and costs. Novel binder systems (e.g., fast-curing polymers or hybrid inorganic-organic binders) have been tested to enable rapid drying of shells [39]. For example, Lin et al. reported a silicone resin–bonded ceramic shell that could be dried much faster than traditional shells while achieving higher strength, thereby potentially shortening the lead time for casting without compromising quality [40].

## 2. Materials and Methods

The samples tested were two types of multi-layered spherical ceramic molds, prepared by the immersion method according to the procedures described in publications [5,6,25]. The samples were made using the dip-and-sprinkle method. Materials of this shape were dedicated to gas permeability measurements, but other tests were also performed on them. Corundum flour (Treibacher Industrie AG, Althofen, Austria) with a particle size of 325 mesh (approx. 30 µm) was used to obtain ceramic funnel masses. The ecological modifier of the fifth layer was walnut shell chips (1 mm to 2 mm). The polymer binder was LUDOX AM30 (Grace, Columbia, MD, USA) containing colloidal SiO_2_. Corundum sand of two particle sizes was used as sprinkles: Corundum 100 (0.1 mm to 0.3 mm) for the first two layers and Corundum 46 (0.3 mm to 0.4 mm) for the structural layers. The funnel masses were mixed in a CAT R50 mechanical mixer (CAT Engineering Office, M. Zipperer GmbH, Ballrechten-Dottingen, Germany) for 14 days at 150 rpm until the slurries stabilized before application. The raw material compositions of the molding compounds and sprinkles are shown in Table 1.

During the research, the corundum meal, corundum sprinkle, and the basic properties of the molding binder used were investigated. Observations of the quartz meal, the sands used for the sprinkle layers and the mold samples were made in a Hitachi SU70 scanning electron microscope (Hitachi High-Tech Corporation, Tokyo, Japan) at an electron beam accelerating voltage of 15 kV.

The polymer binder was characterized by examining its morphology using a Hitachi 5500 scanning electron microscope (Hitachi High-Tech Corporation, Tokyo, Japan), determining the pH and solid phase content (designated as C), and measuring the outflow time from a Zahn cup No. 4, a standard method for viscosity assessment used in precision casting. The reaction of the binders was tested using a Sension-1 Hach pH-meter (Hach Company, Loveland, CO, USA).

Gas permeability was determined in a unique test bench designed and constructed at the Faculty of Materials Science and Engineering of the Warsaw University of Technology.

## 3. Research Results and Discussion

The results of the basic properties of the LUDOX AM30 binder (Grace, Columbia, MD, USA) are presented in Table 2. The binder, characterized by a pH close to neutral and a viscosity similar to that of water (flow time of 6.23 s). The proportion of the solid phase in the form of nano-SiO_2_ particles is typical for molding binders and amounts to 30 wt.%.

**Figure 1:**
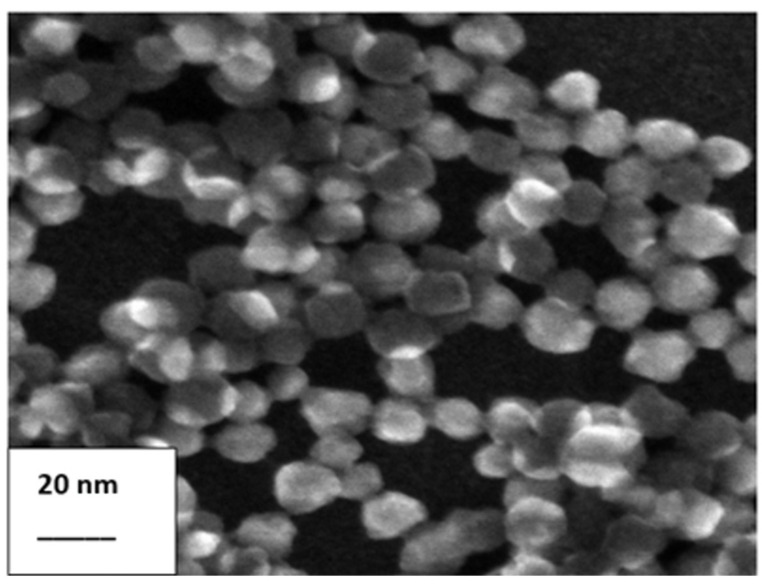
SEM morphology of the LUDOX AM30 binder (Grace, Columbia, MD, USA).

Figure 1 shows an example of the morphology of the LUDOX AM30 binder (Grace, Columbia, MD, USA) obtained using scanning electron microscopy (SEM).

The nano-SiO_2_ particles are characterized by slight agglomeration. The shape of the silica particles is close to spherical, and their diameter does not exceed 20 nm. Figure 1 shows example images of the granular materials used, taken on a Hitachi SU70 scanning electron microscope (Hitachi High-Tech Corporation, Tokyo, Japan).

**Figure 2:**
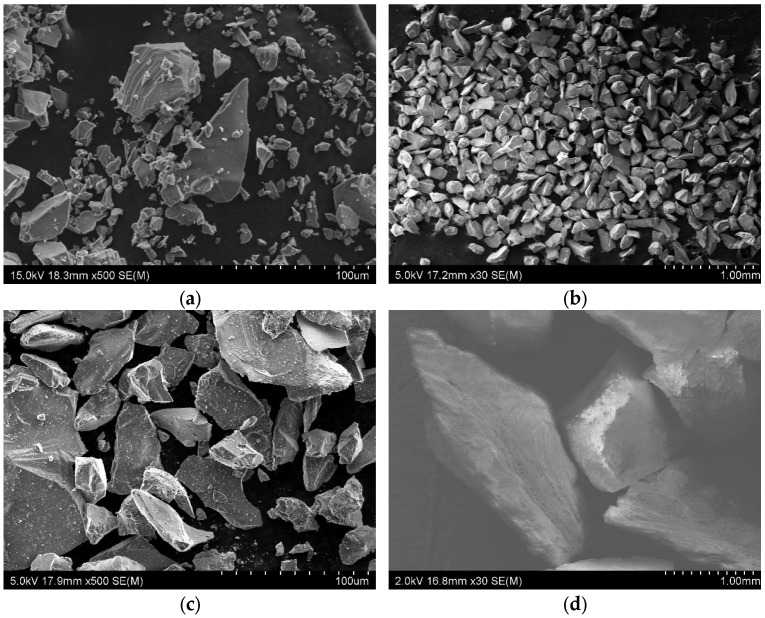
Particle morphology of the powders used to make the mold samples: (**a**) Corundum flour; (**b**) Corundum 100; (**c**) Corundum 46; (**d**) Walnut shell chips.

From the comprehensive microstructural analysis of the solid-phase components conducted using SEM, it is evident that the corundum flour used as the primary fine fraction in the ceramic slurry exhibits a markedly irregular particle morphology (Figure 2a). The particles are angular with sharply defined edges and a high aspect ratio, indicative of a mechanically milled alumina source. This geometry contributes significantly to a higher packing density and mechanical interlocking between adjacent particles in the green state. Similar morphological characteristics are observed for the two granular refractory fractions used as stucco: Corundum 100 (Figure 2b) and Corundum 46 (Figure 2c). Both materials exhibit non-spherical, faceted grains with well-developed crystallographic edges, which is typical for fused and crushed alumina particles. These features enhance the mechanical keying between successive shell layers during the dip-and-sprinkle process, promoting robust adhesion of the stucco to the slurry-coated pattern surface. Walnut shell chips are shown in Figure 2d.

Such morphology is widely accepted in industrial precision casting practice, not only due to its favorable mechanical interlocking but also because it supports adequate capillary suction and binder retention during shell construction. Moreover, the high angularity of these particles creates tortuous pore pathways in the ceramic matrix, which are essential for achieving a balance between gas permeability and thermal stability. Notably, the surface roughness and irregular contact points among the corundum grains help control shrinkage behavior during drying and firing, reducing the incidence of warping or delamination in intermediate layers.

**Figure 3:**
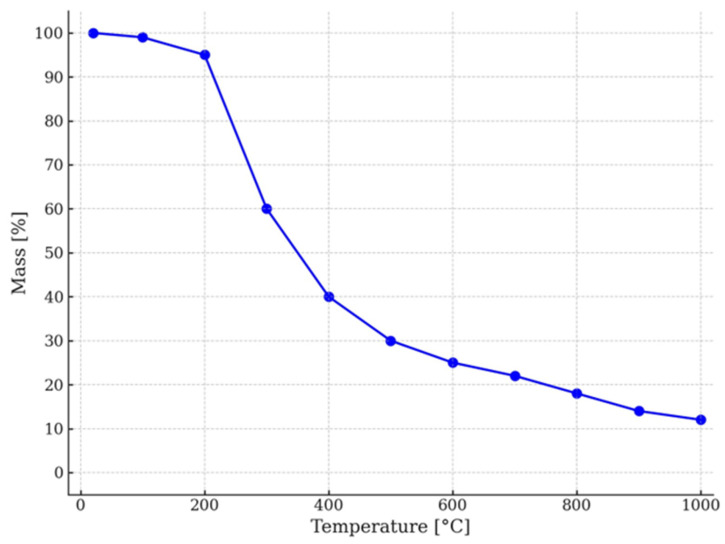
TGA curve of walnut shell chips.

Thermogravimetric analysis (TGA) of the walnut shell chips was performed in an air atmosphere using Q5000 analyzer (TA Instruments, New Castle, DE, USA). Samples were scanned in the temperature range from 20 °C to 1000 °C with a heating rate 10 °C/min. The TGA results of walnut shell chips are shown in Figure 3.

The investigated walnut shell sample exhibited a mass loss of approximately 5% up to 200 °C, attributed to the release of physically and chemically bound water. With increasing temperature in the range of about 200–300 °C, the decomposition of hemicellulose occurs, followed by the thermal degradation of cellulose between 300 °C and 400 °C. At temperatures up to 400 °C, this process is rapid, accounting for a significant weight loss of approximately 60%. During this stage, volatile organic compounds, gases (CO, CO_2_), and carbon are formed. Above 400 °C, a further gradual mass loss is observed, associated with the decomposition of lignin, which occurs over a broad temperature range up to approximately 600–700 °C. This process is slow due to the high thermal stability of lignin. The total mass loss reaches 88%, with ash remaining as the final residue.

**Figure 4:**
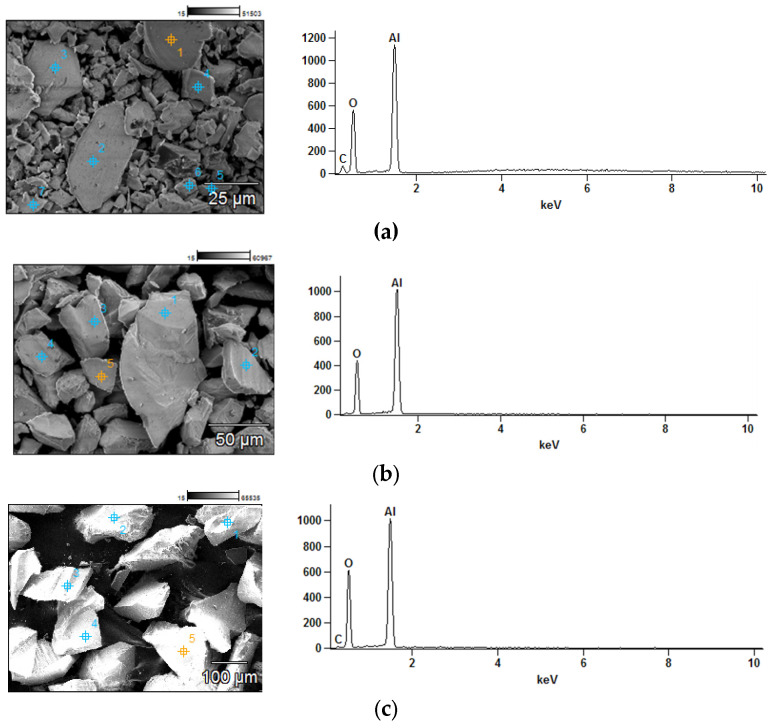

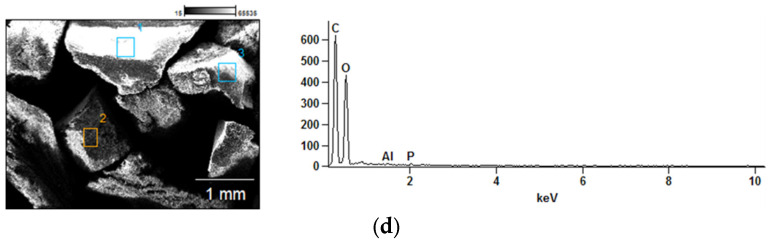
The outcome of the EDS analysis of Corundum 100 within micro-areas: (**a**) Corundum flour; (**b**) Corundum 100; (**c**) Corundum 46; (**d**) Walnut shell chips.

Figure 4 presents the results of the energy dispersive spectroscopy (EDS) analysis performed on corundum samples and walnut shells in selected micro-areas.

The results obtained for all corundum samples confirmed that aluminum (Al) is the principal constituent of the powders. The walnut shell sample represents an organic material of natural origin. It contains carbon (C), oxygen (O), as well as minor amounts of aluminum (Al), phosphorus (P), and silicon (Si).

**Figure 5:**
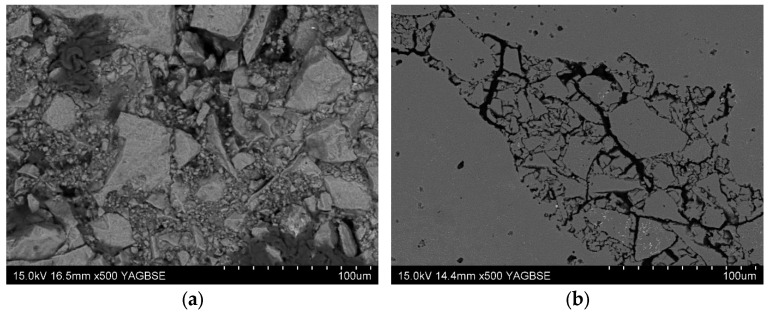

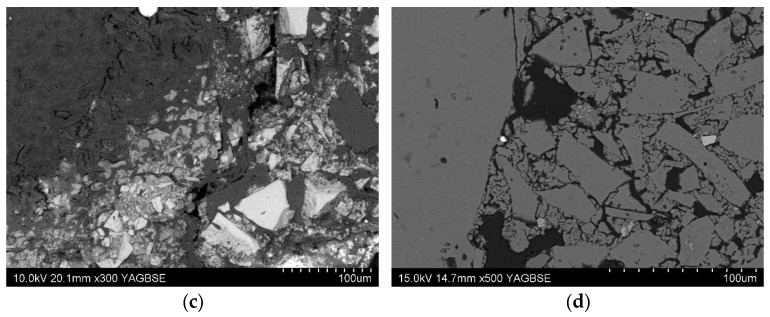
SEM morphology of the fifth layer of ceramic mold samples: (**a**) PW1 sample after soaking at 150 °C; (**b**) PW1 sample after annealing at 950 °C; (**c**) PW2 sample after soaking at 150 °C; (**d**) PW2 sample after annealing at 950 °C.

Detailed SEM micrographs of the fifth structural layer, before and after thermal treatment, offer critical insights into the evolution of the microstructure and its implications for permeability and structural integrity (Figure 5). In the PW1 reference sample, following thermal conditioning at 150 °C (Figure 5a), a heterogeneous and porous network is discernible. Coarse stucco grains (Corundum 46) are embedded in a finer matrix derived from the LUDOX AM30-based slurry (Grace, Columbia, MD, USA). The interface between the coarse and fine particles reveals localized voids and discontinuities, suggesting partial binder shrinkage or incomplete densification during drying. The observed porous structure is characteristic of green shells prepared with colloidal silica binders, which tend to form open gel networks with moderate capillary densification.

Upon high-temperature firing at 950 °C (Figure 5b), the same sample exhibits clear signs of structural degradation in the form of microcracking and interfacial decohesion, particularly at the grain boundaries between the sprinkle corundum and the flour matrix. These cracks predominantly nucleate at the binder-rich zones, implying that the silica sol underwent phase changes (e.g., gel-to-glass transition or sintering-induced shrinkage) that were not fully compatible with the thermal expansion behavior of the refractory components. The mismatch in thermal coefficients and the limited wetting capability of LUDOX AM30 (Grace, Columbia, MD, USA) with highly angular corundum grains may have exacerbated stress concentrations, ultimately leading to defect propagation. Such microstructural flaws, although not immediately catastrophic, can undermine the mechanical strength of the shell and reduce its ability to resist metallostatic pressure during casting.

The modified PW2 sample, incorporating 5 wt.% ground walnut shell chips into the fifth layer, presents a markedly different microstructural evolution. In the pre-fired state at 150 °C (Figure 5c), the presence of coarse, irregular organic inclusions are clearly visible within the ceramic matrix. These walnut shell fragments appear as elongated or flake-like entities with low interfacial bonding to the surrounding ceramic phase. The organic phase is thermally unstable and expected to undergo complete combustion during firing, acting as a fugitive additive. Following high-temperature annealing at 950 °C (Figure 5d), these inclusions are entirely removed, leaving behind large, randomly distributed pores that are often tens of micrometers in diameter.

These voids constitute artificial porosity within the shell structure and serve as microcapillaries for gas evacuation during the casting process. Their stochastic distribution enhances through-thickness gas flow without requiring modification of permeability across all layers of the shell. However, similar to the PW1 sample, the PW2 shell also displays interfacial cracking, particularly at the boundaries where the binder once connected the stucco to the matrix. While the pore-forming strategy successfully introduces targeted permeability enhancement, it also increases the internal surface area and potentially weakens the local matrix integrity. The resultant network of interconnected pores may facilitate outgassing during casting but may also require careful control to ensure that the shell retains adequate mechanical performance under load.

The next stage of the research was gas permeability measurements. The samples obtained by the dip-and-sprinkle method were subjected to a heat treatment of 950 °C for 1 h in a chamber furnace under an air atmosphere to remove the celluloid sphere, on which successive ceramic layers were subsequently applied. The furnace was heated at a rate of 5 °C/min. After reaching the annealing temperature to avoid cracking, the samples were cooled in the furnace.

**Figure 6:**
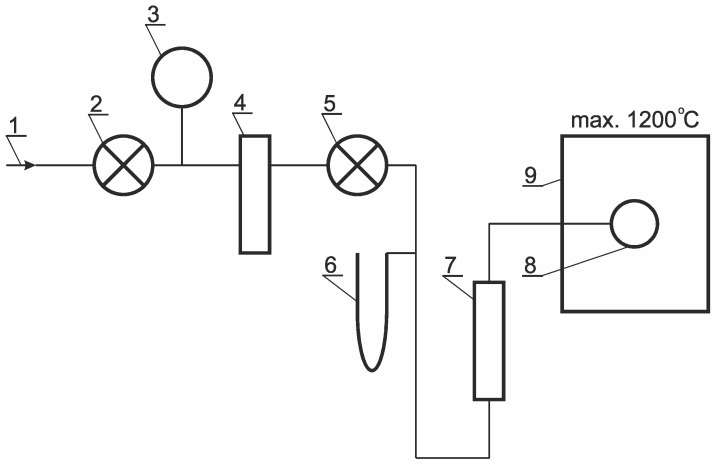
Block diagram of the test station (1—air inlet; 2—membrane valve; 3—dial pressure indicator; 4—air drier; 5—needle valve; 6—column pressure gauge; 7—flowmeter; 8—sample; 9—chamber furnace).

Gas permeability measurements were conducted on a test stand of our own design, which is described in detail in [25]. The design of the measuring station for gas permeability testing in a hot medium is shown in the block diagram in Figure 6. The station consists of a source of compressed air 1, which is a gas cylinder connected to a membrane valve 2 through a reducing valve. The membrane valve 2 is connected to a dial pressure indicator 3 and an air dryer 4. The outlet of the air dryer 4 is connected to a needle valve 5, which precisely regulates the pressure of the air reaching the column pressure gauge 6 and the flowmeter 7. The outlet of the flowmeter 7 is connected to the sample under test 8. The sample 8 is located at the end of the station and is placed in a chamber furnace 9. During the measurement, the air flow is recorded, and its pressure is read from the column pressure gauge 6.

Gas permeability was calculated from the relationship described by Darcy’s law [38,39,41]:

(1) $$K=\frac{M\cdot Q^{\prime }\cdot D}{P\cdot A}$$

where *K* denotes the gas permeability [cm^2^], *M* is the dynamic viscosity of air at a given temperature [(kg/m⋅s) 10^−5^], *D* denotes the thickness of the mold wall [mm], *A* is the mold surface area [mm^2^], *P* represents the air pressure [mm H_2_O] and *Q*′ is the value of air flow at the measurement temperature [cm^3^/min].

The dynamic viscosity of air *M* in the temperature range under study is directly proportional to temperature. For the temperature range of interest, this function takes the form of a linear relationship, so the value of dynamic viscosity for a particular temperature can be directly read out from the device [25].

The value of air flow *Q’* is dependent on the ambient temperature. The measurement is carried out in media with different temperatures using a chamber furnace, so the working medium of air also has different temperatures. With this in mind, a correction must be made for the value of air flow at the measurement temperature described by equation:

(2) $$Q^{\prime }=\frac{Q\cdot T^{\prime }}{T}$$

where *Q*′ denotes the value of air flow at the measurement temperature [cm^3^/min], *Q* is the value of air flow at the room temperature [cm^3^/min], *T*′ denotes the value of the measurement temperature [°C] and *T* is the value of the room temperature [°C].

**Figure 7:**
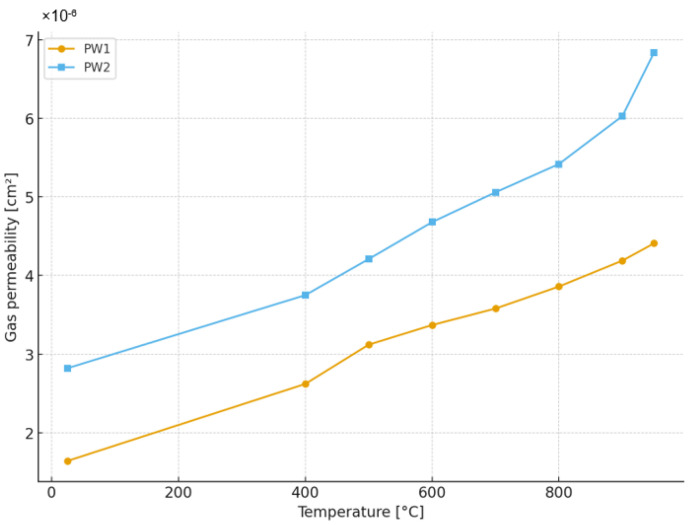
Results of gas permeability measurements of samples PW1 and PW2.

The station enabled gas permeability to be measured at elevated temperatures, allowing for a result as close as possible to the operating conditions of the ceramic mold. Gas permeability tests were conducted in the temperature range of 25 to 950 °C on samples that had been previously subjected to the burnout process. The gas permeability measurement results of the tested casting mold samples are shown in Figure 7.

The experimental results clearly indicate a temperature-dependent increase in gas permeability for both types of ceramic shell mold samples (PW1 and PW2). As the temperature rises from ambient (25 °C) to elevated casting-relevant conditions (950 °C), the permeability of the shells increases by approximately 2.5 times, which is consistent with the expected thermal dilation of the pore network and the concomitant decrease in air viscosity. This thermally activated permeability enhancement is intrinsic to porous ceramic structures and arises due to the combined effects of increased molecular motion, binder pyrolysis residuals, and reduced resistance to gas flow across the intergranular voids.

Notably, the modified sample (PW2), which incorporates a deliberate structural modification in the fifth shell layer through the addition of 5 wt.% walnut shell chips exhibit a consistently higher gas permeability across the entire temperature range. This enhancement can be directly attributed to the combustion of walnut shell particles during firing, which leaves behind a network of stochastically distributed macrovoids. These voids, typically tens of micrometers in size, serve as preferential pathways for gas transit, substantially reducing the resistance to flow through the shell. Importantly, these pores are concentrated within a single intermediate layer, yet they exert a cascading influence on the permeability of the entire multilayer shell, enabling improved air displacement not only locally but also through adjacent structural zones.

Beyond the established parameters such as particle morphology, binder type, and layer thickness, this study underscores the significant role that localized structural modification can play in tuning shell permeability. The incorporation of organic waste-based fillers, such as walnut shell particulates, into the ceramic slurry introduces a sustainable and cost-effective strategy for engineering pore architecture. Quantitative analysis reveals that the use of walnut shell chips increases gas permeability by approximately 36% at elevated temperatures and up to 42% at room temperature, when compared to the unmodified reference sample. These improvements are substantial and validate the effectiveness of the pore-forming approach.

A key insight derived from this work is the understanding that permeability control does not necessarily necessitate a comprehensive redesign of all shell layers. Instead, modifying a single strategically selected layer—specifically, one that does not compromise structural integrity or surface finish—can yield measurable and reproducible improvements in gas transport properties. This modular approach to permeability enhancement simplifies implementation and minimizes risks to shell strength, making it a practical solution for industrial foundries aiming to optimize process reliability without overhauling the entire shell-making protocol.

From an application standpoint, the findings of this study suggest that ceramic shell molds of the PW2 configuration are promising candidates for deployment in precision casting operations, particularly for turbine or aerospace-grade components. However, before industrial adoption, it is essential to validate the mechanical performance, erosion resistance, and thermal behavior of the modified shells under real casting conditions. This would include liquid metal filling trials under vacuum or inert gas environments, mechanical testing of shells post-firing, and post-casting evaluation of the surface quality and microstructure of the resulting metallic components.

## 4. Results and Analysis

The experimental investigations conducted on the multilayer ceramic shell systems—namely the reference sample (PW1) and the modified sample incorporating walnut shell particles (PW2)—highlight key structure–property relationships pertinent to investment casting technology. Central to the analysis is the role of pore-forming strategies, particle–binder interactions, and thermal processing in shaping the gas permeability and microstructural evolution of the shell molds. The results clearly show that a targeted modification of a single intermediate layer (fifth layer) using 5 wt.% walnut shell chips can significantly enhance the permeability of the ceramic molds while maintaining the structural logic of the multilayer system.

The increase in gas permeability with rising temperature for both samples aligns well with fundamental gas flow theory in porous materials, where the Knudsen diffusion regime transitions toward viscous flow with increasing temperature and decreasing gas viscosity. Similar thermal enhancement trends were reported in other ceramic mold systems employing colloidal silica binders and high-purity corundum aggregates [25], supporting the reliability of the experimental methodology.

More critically, the superior permeability of the PW2 samples, achieved via thermal burnout of lignocellulosic particles, aligns with recent studies exploring the use of biodegradable pore-forming agents. Pattnaik and Sutar [37] demonstrated that the addition of 4 wt.% polyethylene wax powder in the secondary layer of investment molds increased shell permeability by up to 30% with marginal reductions in strength. Similarly, in a study by Pattnaik and Sutar [36], the inclusion of 5 wt.% sawdust led to an increase in gas permeability of approximately 35–38%, with large interconnected pores forming after pyrolysis. The current study’s finding of a 36–42% permeability increase for the PW2 configuration is consistent with these values and supports the idea that organic fillers of controlled granulometry can serve as effective pore formers.

Notably, the walnut shell filler used in this work differs from traditional synthetic pore-formers by offering a more sustainable, biodegradable, and non-toxic alternative, as also advocated in recent circular economy research [39]. Moreover, the irregular and angular morphology of the walnut shell chips led to the formation of randomly distributed pores with a wide size distribution (tens of micrometers), which enabled multidirectional venting of process gases—a feature crucial in mitigating blowhole formation and entrapped gas defects during high-precision alloy casting [27,38].

The presence of microcracks and interfacial decohesion in both PW1 and PW2 samples after firing, particularly at the grain–binder interface, raises concerns about thermal compatibility between the LUDOX AM30 binder (Grace, Columbia, MD, USA) and angular corundum grains. This phenomenon is consistent with observations by Wiśniewski et al. [25], who reported that the gelation behavior of silica sol binders can lead to localized stresses and cracking upon sintering, especially when the slurry exhibits poor wetting characteristics or a mismatch in binder shrinkage. These findings suggest that future optimization could involve hybrid or polymer-modified binders (e.g., colloidal silica with poly (vinyl alcohol)), which have demonstrated enhanced green strength and reduced crack propagation in similar systems [23].

The microstructural observations from SEM analysis confirm the hypothesis that the pores introduced by walnut shell burnout dominate the gas flow behavior through the shell. Unlike PW1, where porosity arises mainly from intergranular gaps and packing imperfections, the PW2 shells contain deliberate macroporosity with higher aspect ratios and wider necks, as also seen in studies on ABS powder and needle coke-modified shells [27]. Importantly, the localized concentration of pores in one structural layer (rather than homogenously across all layers) did not compromise shell integrity, confirming that permeability tuning can be selectively implemented without overhauling the entire shell composition.

Comparing the present results with those of Kanyo et al. [11], who provided a comprehensive overview of investment casting shells for superalloy components, it becomes evident that modern shell design must simultaneously address contradictory requirements: mechanical strength, thermal resistance, permeability, and process economy. The shell modification technique used in this study—targeted pore engineering via bio-additive burnout—achieves a favorable compromise among these constraints and is well-suited for components cast under vacuum or inert conditions, where gas evacuation paths are critically important.

The study contributes to the growing body of work emphasizing sustainable materials engineering in high-value manufacturing sectors. The use of ground walnut shell—an agricultural byproduct—mirrors the valorization strategies outlined by Koralnik [38], where spent ceramic shell waste was repurposed into a refractory feedstock, thereby closing the materials loop. In this light, the present work not only enhances performance but also reinforces the movement toward environmentally responsible foundry practices.

The uniqueness of walnut shells lies in the combination of hardness, natural origin, versatility, and environmental friendliness. As a result, they find applications in industry, cosmetics, energy production, horticulture, and even natural medicine. Walnut shells are a completely natural and biodegradable raw material [42,43,44]. They can be utilized as a pore-forming agent in ceramic products. Their unique structure and chemical composition ensure that, after proper processing, they become an effective and ecological source of porosity [45,46]. In ceramic materials, walnut shells undergo almost complete burnout during firing. Walnut shells have been found to be a unique and interesting material for precision casting.

## 5. Conclusions

This study investigated the influence of a biodegradable pore-forming additive—ground walnut shell particles—on the microstructure and gas permeability of multilayer ceramic shell molds prepared via the conventional dip-and-stucco method.

- Ceramic shell molds fabricated from corundum powders (Corundum 46 and Corundum 100) with a colloidal silica binder (LUDOX AM30) exhibited temperature-dependent permeability, which increased significantly at elevated temperatures.
- Selective modification of the fifth layer by introducing 5 wt.% walnut shell chips produced a substantial permeability improvement compared with the unmodified reference shell.
- Upon firing at 950 °C, the walnut shell particles decomposed completely, leaving behind large, irregular pores that enhanced gas flow paths.
- Quantitative testing showed that PW2 achieved 42% higher permeability than PW1 at room temperature and 36% higher at 950 °C.
- Crucially, the modification of only one internal layer proved sufficient to achieve functional permeability control, avoiding risks associated with altering all layers.
- Walnut shells—abundant, biodegradable, and low-cost—proved to be an effective and sustainable pore-forming agent for ceramic molds.
- The results demonstrate strong potential for industrial application, although further validation under real casting conditions is required to confirm mechanical robustness and casting performance.
- Overall, the findings support the integration of sustainable, waste-derived additives into advanced foundry technology, contributing both to defect reduction and to circular economy objectives.

## Figures and Tables

**Table 1:** Basic raw material compositions of the samples produced.

Sample	Layer	Molding Compound	Sprinkle
PW1	1 and 2	LUDOX AM30 and corundum flour	Corundum 100
	3 to 6	LUDOX AM30 and corundum flour	Corundum 46
	7	LUDOX AM30 and corundum flour	–
PW2	1 and 2	LUDOX AM30 and corundum flour	Corundum 100
	3 and 4	LUDOX AM30 and corundum flour	Corundum 46
	5	LUDOX AM30 and corundum flour with 5 wt.% walnut shell chips	Corundum 46
	6	LUDOX AM30 and corundum flour	Corundum 46
	7	LUDOX AM30 and corundum flour	–

**Table 2:** Basic properties of the polymer binder used.

Molding Binder	pH	C [%]	Outflow Time from Zahn Cup No. 4 [s]
LUDOX AM30	8.74	30.0	5.69

## Data Availability

The original contributions presented in this study are included in the article. Further inquiries can be directed to the corresponding author.
